# Early Use of Liraglutide for the Treatment of Acute COVID-19 Infection: An Open-Label Single-Center Phase II Safety Study with Biomarker Profiling

**DOI:** 10.3390/idr17010005

**Published:** 2025-01-10

**Authors:** Eloara V. M. Ferreira, Rudolf K. F. Oliveira, Reinaldo Salomao, Milena K. C. Brunialti, Martyella B. A. Cardoso, Chien-nien Chen, Lan Zhao, Colm McCabe

**Affiliations:** 1 Division of Respiratory Diseases, Department of Medicine, Federal University of São Paulo (UNIFESP), São Paulo 04038-901, Brazil; eloara.ferreira@unifesp.br (E.V.M.F.); rudolf.oliveira@unifesp.br (R.K.F.O.);; 2 Division of Infectious Diseases, Department of Medicine, Federal University of São Paulo (UNIFESP), São Paulo 04038-901, Brazilmilenacbrunialti@gmail.com (M.K.C.B.); 3 Department of Experimental Medicine, Hammersmith Campus, Imperial College, London SW7 2BX, UKl.zhao@imperial.ac.uk (L.Z.); 4 Royal Brompton Hospital, Part of GSTT NHS Foundation Trust, London SW3 6NP, UK; 5 National Heart and Lung Institute, Imperial College, London SW3 6NP, UK

**Keywords:** COVID-19, GLP-1 agonist, CD147

## Abstract

Background: Glucagon-like peptide-1 (GLP-1) agonists are an existing treatment option for patients with insulin-resistant states, which elicit further pleiotropic effects related to immune cell recruitment and vascular inflammation. GLP-1 agonists downregulate the cluster of differentiation 147 (CD147) receptor, one of several receptors for the SARS-CoV-2 spike protein that mediate viral infection of host cells. Methods: We conducted an open-label prospective safety and tolerability study including biomarker responses of the GLP-1 agonist Liraglutide, administered for 5 days as an add-on therapy to the standard of care within 48 h of presentation in a cohort of 13 patients hospitalized with COVID-19 pneumonia. Biomarker responses were compared in patients admitted to critical care and those not requiring critical care admission (non-critical group). Results: Liraglutide (0.6 mg, subcutaneously) was well tolerated by all patients and all patients were alive 30 days after diagnosis. Plasma soluble CD147 levels were reduced in the non-critical patient group at day 5 in contrast to critical care-treated patients, who demonstrated an increase in soluble CD147 levels between day 0 and day 5. Patients with milder COVID-19 pneumonia severity also demonstrated improvement in echocardiographic parameters of right and left ventricular function, reduction in plasma Troponin levels, increased CD147 expression on T lymphocytes, and reduction in plasma IL-8. Conclusions: This first-in-disease use of the GLP-1 agonist Liraglutide demonstrates its safety and tolerability in an unselected cohort of patients hospitalized with COVID-19 pneumonia across a range of clinical severities.

## 1. Introduction

The earliest phase of the COVID-19 pandemic included significant numbers of hospitalized patients at risk of respiratory failure and progressive end-organ dysfunction. Since then, the treatment for acute COVID-19 pneumonia has evolved to include several novel therapeutic strategies, the prioritization of which depends on factors including, among others, disease severity, the degree of end-organ involvement, and underlying patient comorbidities [1]. In patients with an elevated premorbid risk of COVID-19 infection, such as more elderly patients or those with diabetes, obesity, or cardiovascular disease, mortality has remained disproportionately high [2,3]. Specific patient subgroups with different disease severities continue to demonstrate variation in treatment response [4]. Supportive treatments and novel therapeutic management strategies for this population are key to addressing adverse clinical courses in this patient group.

Glucagon-like peptide-1 (GLP-1) agonists are a treatment option for patients with insulin resistance and cardiovascular disease, demonstrating improvement in a broad range of cardiovascular outcomes and metabolic endpoints in a series of landmark studies [5,6,7]. Mediated through the activation of the GLP-1 receptor, GLP-1 agonists elicit a variety of pleiotropic effects related to the reduction in immune cell recruitment, vascular adhesion molecule expression, and oxidative stress [8,9]. In addition, the favorable effects of GLP-1 agonists, which improve myocardial energy substrate utilization in experimental heart failure models and target the insulin-resistant state associated with hospitalized patients with COVID-19 pneumonia, support a clinical rationale for their use in patients with more severe infection, where cardiovascular complications occur most frequently [10,11].

The cluster of differentiation 147 (CD147) receptor, a highly glycosylated Type 1 transmembrane glycoprotein of the immunoglobulin superfamily and prominent matrix metalloproteinase (MMP) inducer, is one of several receptors for the SARS-CoV-2 spike protein that mediate viral infection of host cells [12] and may be downregulated by the GLP-1 agonist Exenatide. This is hypothesized to occur via the transition of membrane-bound CD147 from a high to a low glycosylation status [13]. Furthermore, CD147 expression is upregulated in a range of diseases, including thrombosis, diabetes, and obesity [14], and in a cohort of patients with acute COVID-19 infection, we have demonstrated its association with increased disease severity and poorer prognosis [15]. Transcriptomic databases show an age-related increase in CD147 expression on vascular endothelial cells [16]. This suggests a potential mechanism for the observed increase in the risk of severe COVID-19 infection in patients over 40 years of age and in those with Type 2 diabetes, in whom CD147 expression on circulating immune cells is increased in response to high blood glucose levels [17].

We hypothesized that the GLP-1 agonist Liraglutide would have a favorable effect on CD147-mediated vascular inflammation in acute COVID-19 pneumonia. Here, we report the results of a prospective evaluation of its use and administration as an add-on therapy to the standard of care within 48 h of presentation in a cohort of 13 patients hospitalized with COVID-19 pneumonia. Directed primarily at safety and tolerability within an open-label design that included the evaluation of established cardiac biomarkers of COVID-19 severity, we collected clinical outcome data at hospital discharge and after 30 days in all patients. Biomarker responses in plasma levels of soluble CD147 (a by-product of CD147 surface membrane protein cleavage) and CD147 positive lymphocyte subpopulations at day 0 and day 5 were analyzed following treatment with Liraglutide.

## 2. Materials and Methods

We performed a prospective single-center open-label phase II study in adults (≥18 years old) hospitalized due to COVID-19 pneumonia at Hospital Sao Paulo, the Teaching Hospital of the Federal University of São Paulo (Unifesp), Brazil, between February and April 2021 to test the safety and tolerability of Liraglutide as an add-on therapy to the standard of care. This clinical trial was approved by Unifesp’s Ethics Committee and by the Brazilian National Research Ethics Commission (CONEP) and was conducted following good clinical practice guidelines. Written informed consent was obtained from all study participants (CAAE 38657420.2.0000.5505, CONEP 4.416.704), and the protocol was registered at the Brazilian Clinical Trials Registry (RecBec RBR-8vyvnmb).

Patients were included in this study within 48 h after hospital admission and required a confirmed diagnosis of COVID-19 using a reverse transcriptase–polymerase chain reaction (RT-PCR) assay on nasopharyngeal swab samples. The exclusion criteria at study entry (day 0) were an estimated life expectancy of less than 48 h derived from the clinical opinion of one or more members of the study team [18] and standard contraindications for GLP-1 agonist therapy. At recruitment, all included patients received a predefined fixed dose of Liraglutide (0.6 mg, subcutaneously, once a day) for 5 days. They were followed clinically every day for 5 days, and clinical data were recorded on day 0 (baseline, before the first dose of Liraglutide) and day 5 (after the 5th dose of Liraglutide). After entry into this study and according to the clinical care setting needed in the subsequent 5 days, patients were classified as non-critical (ward admission with oxygen requirement by a nasal catheter or face mask < 5 L/min) or critical (ICU admission requiring high flow oxygen, non-invasive ventilation, or mechanical ventilation). All patients were followed until 30 days after study entry for adverse event recording. At study inclusion, venous blood was drawn from patients on day 0 and day 5 and processed according to standard laboratory techniques. Additionally, twenty milliliters of blood were collected in K3 EDTA tubes (Greiner bio-one Americana, Sao Paolo, Brazil) for Peripheral Blood Mononuclear Cell (PBMC) isolation, and the plasma was separated for cytokine/chemokine analysis.

The primary outcome was the safety and tolerability of Liraglutide as an add-on therapy to the standard of care in hospitalized patients with confirmed COVID-19 pneumonia. Exploratory endpoints were evaluated at baseline (day 0) and at end of this study (day 5) and included the change in soluble CD147 and plasma cytokines/chemokines.

Tabulated data are presented as mean ± SD unless otherwise stated. Comparison between two groups was performed using a *t*-test for continuous data or a Chi-squared test for categorical data. A paired *t*-test was conducted to compare the effect of treatment before (day 0) and after (day 5) the use of Liraglutide. A *p*-value < 0.05 was considered significant. The statistical analysis was performed using SPSS software (version 19; IBM Company, Armonk, NY, USA).

## 3. Results

Fifteen consecutive patients were prospectively included in this study. Two patients withdrew their consent before this study ended, and one patient had an early hospital discharge before completing this study (i.e., prior to day 5). Therefore, the laboratory study samples were obtained from 13 patients, 12 with complete data collection (i.e., day 0 and day 5) and one subject with only the baseline data (i.e., day 0). The patients’ baseline characteristics are presented in Table 1 and Supplementary Materials.

**Figure 1:**
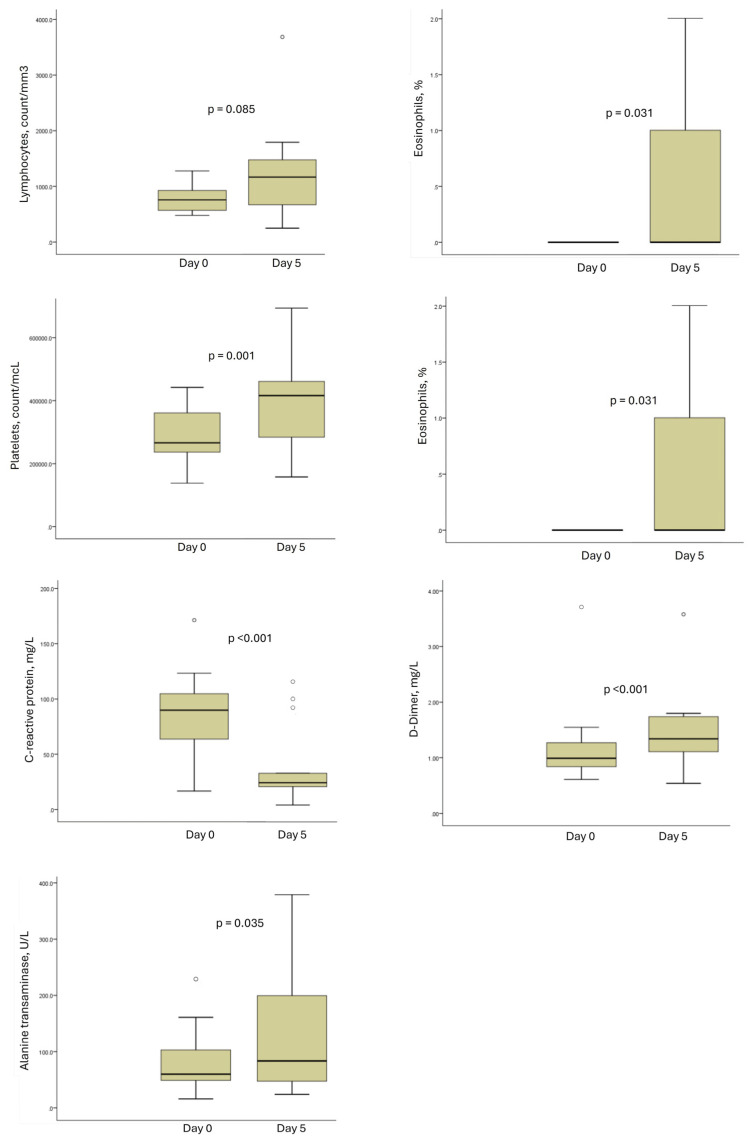
Significant changes in clinical parameters from peripheral blood in all study patients in response to 5 days administration of Liraglutide (data recapitulated from Table 2).

This study demonstrated that the addition of Liraglutide (0.6 mg, subcutaneously) to the standard of care was well tolerated, with no occurrence of adverse events. All patients survived until hospital discharge and were alive 30 days after diagnosis. Relevant safety endpoints demonstrated no hypoglycaemic events or metabolic perturbation associated with the administration of Liraglutide (Table 2), with significantly altered variables displayed in Figure 1. Evaluating patients according to their requirement for critical care admission, cardiac biomarker (Troponin) and echocardiographic data (LV ejection fraction and right ventricular fractional area change) demonstrated potential improvement in cardiac function in patients with milder disease severity over the course of this study (Table 3 and Supplementary Materials, Table S1). Further detailed safety endpoints are provided in Table S2 (Supplementary Materials) with individual patient data presented in Table S3 (Supplementary Materials).

### 3.1. CD147 Expression on Lymphocytes and Soluble Plasma Levels

**Figure 2:**
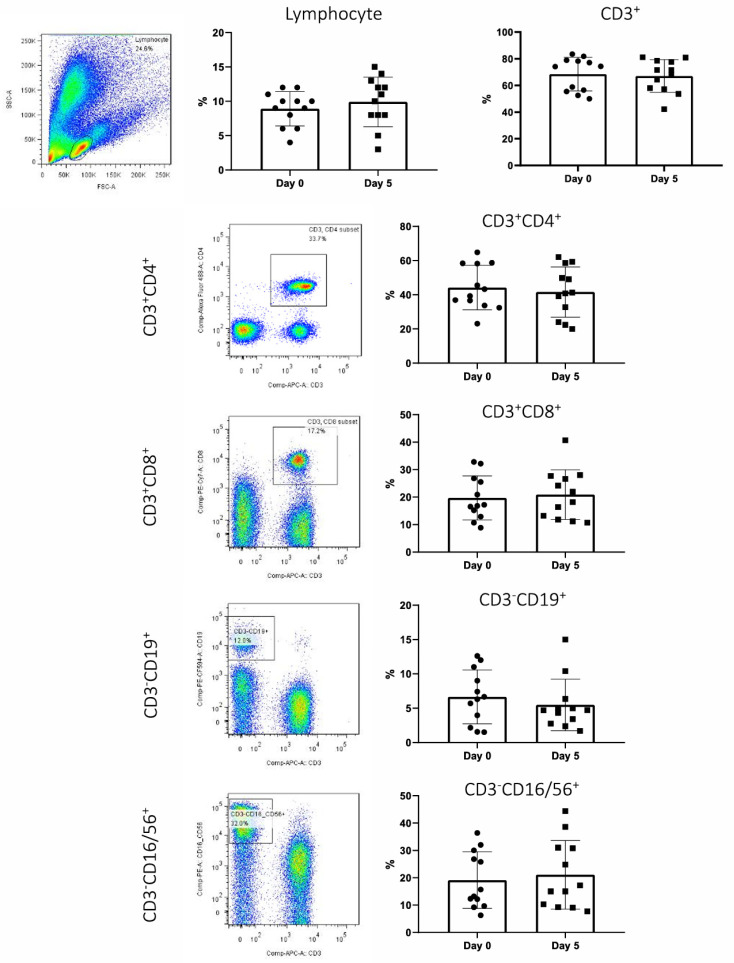
Fluorescence-activated cell sorting (FACS) data showing changes in mean geometric fluorescence intensities of CD147 positive lymphocyte subpopulations (CD4, CD8, CD19, and CD56 positive) between day 0 and day 5 after treatment with Liraglutide (green/red colour represent greater fluorsecence intensity). No significant changes were observed in fluorescence intensities of CD147 positive lymphocytes across the whole study group following the administration of Liraglutide.

The composition of circulating lymphocytes harvested from 12 patients at day 0 and after Liraglutide treatment at day 5 was analyzed by FACS, with the data presented in Table 4 and Figure 2. No significant changes were found between day 0 and day 5 when comparing the proportion (%) of total lymphocytes, T (CD3+), T help (Th, CD3+CD4+), T cytotoxic (Tc, CD3+CD8+), B (CD3-CD19+), and natural killer (NK, CD3-CD16/56+) lymphocytes (Table 4).

An increase in CD147 expression was found on T lymphocytes, representing both Th and Tc lymphocytes in the total study sample. We noted that increased expression of CD147 on both Th and Tc lymphocytes was only seen in the non-critical cohort. However, patients admitted to critical care had a relatively higher CD147 expression level on all lymphocyte subgroups at day 0 compared to patients with milder disease, with no change in the expression level after Liraglutide treatment at day 5. There were no significant changes in plasma soluble CD147 levels between day 0 and day 5 in the total study sample. However, plasma soluble CD147 levels were reduced in the non-critical patient group at day 5, in contrast to critical care-treated patients who demonstrated an increase in soluble CD147 levels between day 0 and day 5 (Table 4).

### 3.2. Plasma Levels of Circulating Inflammatory Mediators

Cytometric Bead Array showed that the critical care patient group presented with higher plasma IL-6, IL-8, and IL-10 levels at day 0. IL-6 and IL-10 levels were significantly reduced at day 5 after Liraglutide treatment compared to day 0 in the overall study sample, while IL-8 levels decreased only in the non-critical care patient group. ELISA assessment of plasma matrix metallopeptidase (MMP)-2 and -9 levels demonstrated no significant changes between day 0 and day 5, regardless of clinical severity. An increase in MMP-9 was found in non-critical-care-treated patients (Table 4).

## 4. Discussion

In the present work, we describe findings from a prospective safety and tolerability study of GLP-1 agonist Liraglutide administered subcutaneously once daily at a dose of 0.6mg, in addition to the standard of care, which evaluated soluble CD147 levels, cytokine levels, and CD147 lymphocyte expression in patients hospitalized with COVID-19 pneumonia. In a cohort of patients, all of whom survived until hospital discharge, Liraglutide was well tolerated, with no adverse events. Furthermore, there was no evidence of early adverse effects on cardiac function based on cardiac biomarker and echocardiographic parameters measured at day 0 and day 5. Within the 5-day study period, we documented trends in plasma soluble CD147 and cytokine levels, as well as the CD147 expression levels in lymphocyte subpopulations according to disease severity at presentation. To our knowledge, this is the first reported use of the GLP-1 agonist Liraglutide as an add-on therapeutic intervention in hospitalized patients with COVID-19 pneumonia and supports a rationale for further investigation of the role of CD147 in COVID-19 pathogenesis and its pharmacological modulation relevant to COVID-19 treatment.

The relevance of CD147 receptor function to COVID-19 pathogenesis is supported by several factors, in addition to our results regarding GLP-1-mediated receptor deglycosylation. CD147 demonstrates ubiquitous cell surface expression, which includes the vascular endothelium and T lymphocytes and functions as an alternative SARS-CoV-2 receptor in angiotensin-converting enzyme 2 (ACE2)-deficient cells [12]. Furthermore, studies have reported an association with SARS-CoV-2 infectivity, which correlates with CD147 expression levels by target cells [12,19]. Human T cells may be infected with SARS-CoV-2 pseudovirus in a dose-dependent manner, which is inhibited by the human monoclonal anti-CD147 antibody (Meplazumab). These pre-clinical findings align with both an early phase study of Meplazumab showing normalization of lymphopenia and accelerated SARS-CoV-2 viral clearance, in addition to a recent Phase II/III randomized trial of severe COVID-19 pneumonia, which demonstrated that, compared to the placebo, Meplazumab reduced mortality and increased both the proportion of patients alive and discharged without supplemental oxygen and the proportion of patients who achieved sustained clinical improvement [20,21].

Pre-clinical data on the CD147 surface membrane receptor show that it occurs in a highly glycosylated form, a potent MMP inducer interacting with several downstream extracellular and intracellular partners. GLP-1 agonism downregulates the glycosylation status of CD147 receptor function via a transition to a lower glycosylated state [13]. Soluble CD147, measured in our clinical study, may be derived either via cell secretion as a full-length protein within microvesicles or more commonly via the cleavage of the extracellular portion of the membrane-bound CD147 receptor mediated by MMPs or ADAM13. Soluble CD147 expression differed according to the clinical severity of COVID-19 infection in our study, with a reduction in soluble CD147 in patients between day 0 to day 5 in patients not requiring critical care. Although within the limitations of our study design, these changes cannot be directly attributed to Liraglutide or changes in membrane-bound CD147, it is conceivable that different levels of vascular inflammation or T cell activation in patients with milder and more severe disease may have influenced soluble CD147 expression.

Our study aligns with recent evidence supporting a potential role for GLP-1 agonist use in infectious and inflammatory conditions. In the critical care setting, hyperglycemia may be associated with worse outcomes, suggesting that therapies that can control blood glucose with a reduced risk of hypoglycemia may be beneficial. In sepsis, GLP-1 agonists can further regulate the immune response, downregulating inflammatory factors associated with vascular homeostasis and platelet adhesion, with potentially protective effects on organ function [22]. We observed a potentially favorable influence on cardiac biomarkers in patients with milder COVID-19 pneumonia, which was not explained by a reduction in systemic blood pressure, a known effect of GLP-1 agonist therapy. Data from the recently published SELECT study, which focused on an obese population group, were used to assess the effect of Semaglutide on all-cause death, cardiovascular death, and non-cardiovascular death, including events attributed to COVID-19 infection [23]. Here, among participants who developed acute illness, fewer participants treated with Semaglutide vs. the placebo suffered from serious adverse events (232 vs. 277 respectively; *p* = 0.04) or died from the infection (43 vs. 65; HR: 0.66; 95% CI: 0.44–0.96). Perhaps relevant to our observations of a potential cardiovascular benefit of Liraglutide, among the deaths reported in patients who acquired COVID-19 infection, fewer were attributable to cardiovascular causes in patients who received Semaglutide.

The response in CD147 expression on lymphocytes following treatment with Liraglutide in our study showed no overall change between day 0 and day 5 across the whole cohort. However, patients not requiring critical care demonstrated upregulated CD147 expression in both Th and Tc cell populations, in contrast to more severely affected patients. Early COVID-19 studies have suggested reduced circulating lymphocyte numbers (lymphopenia) to be a hallmark feature of severe COVID-19 pneumonia requiring hospitalization. In particular, CD4 positive T cell frequencies are significantly reduced compared to those with milder disease or healthy controls, suggesting an acquired dysregulation of T cell homeostasis in more severe disease. While no significant changes were found between day 0 and day 5 when comparing the proportion (%) of total lymphocytes, T cells, B cells, and NK lymphocytes, CD147 was expressed on all the lymphocyte subpopulations. Here, we observed higher baseline CD147 expression in lymphocyte subpopulations in patients requiring critical care, consistent with the data from our previous retrospective cohort study, where soluble CD147 levels were related to disease severity and survival. On the other hand, upregulated CD147 expression on T lymphocytes in patients with milder disease between day 0 and day 5 raises the possibility of modulation of CD147 lymphocyte expression by Liraglutide in a milder range of disease severity, allowing the activation of T cell function during recovery from COVID-19 infection.

Our clinical study had several limitations, the first one being its size and lack of placebo control. Therefore, our findings do not permit therapeutic recommendations and should be regarded as preliminary. Our study was also conducted in a small cohort of patients over a short duration, which was required to increase recruitment feasibility. However, this may have introduced potential variation in soluble CD147 expression levels, also limiting the conclusions that can be drawn. On a practical level, study inclusion criteria required blood samples to be drawn from patients at day 0 and day 5, which may have reduced the chance of observing the proposed effect. Due to the limitations around sample processing and available resources, no analysis could be undertaken on membrane-bound CD147 glycosylation and its relationship to soluble CD147 levels. In favor of our clinical approach, Liraglutide is more widely accessible among current GLP-1 agonists, with a favorable cost compared to newer GLP-1 agonists. Its once-daily frequency of administration and shorter half-life compared to other GLP-1 agonists arguably confer less risk in patients susceptible to progressive organ dysfunction. This supports the potentially greater applicability of Liraglutide in future studies examining GLP-1 agonist use against COVID-19 and sepsis more generally. In conclusion, we report, to our knowledge, the first-in-disease use of the GLP-1 agonist Liraglutide in COVID-19 pneumonia and demonstrate safety and tolerability in an unselected cohort of patients hospitalized with a range of clinical severities.

## Figures and Tables

**Table 1:** Patients’ baseline characteristics of hospitalized with COVID-19 pneumonia included in the prospective proof-of-concept clinical study using GLP-1 (*n* = 13).

Age, years	55 ± 9
Male gender, *n* (%)	10 (7)
**Comorbidities, *n* (%)**	
Body mass index > 25 kg/m^2^	6 (46)
Systemic hypertension	5 (38)
Current/former smoker	4 (31)
Diabetes	4 (31)
Autoimmune disease	2 (15)
Left heart disease	1 (8)
Asthma	1 (8)
**Symptom, *n* (%)**	
Fever	10 (77)
Cough	10 (77)
Dyspnea	9 (69)
Myalgia	9 (69)
Anosmia	6 (46)
Dysgeusia	5 (38)
Fatigue	3 (23)
Diarrhea	3 (23)
Headache	3 (23)
Nasal congestion	2 (15)
Nausea/vomiting	2 (15)
**Clinical status at admission**	
4C Score, points	7.5 ± 2.4
Lung computerized tomography scan, *n* (%)	5 (38)
**Hospitalization data**	
**Days, median [interquartile range]**	
From symptoms to diagnosis	7 [4–9]
From diagnosis to hospitalization	1 [4–6]
In the intensive care unit	11 [5–23]
In hospital	15 [8–30]
**Treatment, *n* (%)**	
Systemic corticosteroids	13 (100)
Macrolides	5 (38)
Prophylactic anticoagulation	11 (85)
Therapeutic anticoagulation	2 (15)
**Follow-up data, *n* (%)**	
Admission to the intensive care unit	7 (54)
Mechanical ventilation	2 (15)
Clinical improvement during the study period	7 (54)
Deaths	0 (0)

Data are presented as *n* (%), mean ± SD, or median [interquartile range].

**Table 2:** Safety endpoints at baseline and after five days of administration of Liraglutide in hospitalized COVID-19 patients (*n* = 12).

	Day 0	Day 5	*p*-Value
**Clinical data**			
Respiratory rate, ipm	28 [22–29]	20 [19–23]	0.105
SpO_2_, %	94 [92–96]	93 [92–94]	0.245
Heart rate, bpm	84 ± 22	91 ± 15	0.280
Systolic blood pressure, mmHg	124 ± 16	130 ± 18	0.287
Diastolic blood pressure, mmHg	81 ± 16	79 ± 14	0.774
Temperature, Celsius	36.5 [35.9–37.1]	36.3 [36–37]	0.843
**Laboratory results**			
Hemoglobin, g/dL	14.0 ± 16	13.8 ± 1.4	0.674
Hematocrit, %	42 ± 4	41 ± 3	0.589
Leukocytes, count/mm^3^	9096 ± 3547	9712 [8060–11,360]	0.275
Neutrophils, %	86 [83–88]	83 [79–89]	0.131
Lymphocytes, count/mm^3^	757 [562–996]	1167 [632–1499]	**0.085**
Lymphocytes, %	10 [7–12]	11 [8–12]	0.220
Eosinophils, %	0 [0–0]	0 [0–1]	**0.031**
Platelets, count/mcL	284,923 ± 102,743	408,154 ± 162,413	**0.001**
Urea, mg/dL	56 [30–66]	42 [35–55]	0.402
Creatinine, mg/dL	0.8 [0.7–1.1]	0.9 [0.8–1.0]	0.401
Sodium, mEq/L	137 ± 3	136 ± 5	0.261
Potassium, mEq/L	4.3 ± 0.5	4.7 ± 0.5	**0.029**
C-reactive protein, mg/L	89.9 [62.6–112.6]	24.3 [19.7–62.5]	**<0.001**
Lactic dehydrogenase, U/L	422 ± 119	409 ± 138	0.701
D-Dimer, mg/L	1.03 ± 0.30	1.51 ± 0.90	**0.085**
Fibrinogen, mg/dL	639 ± 80	583 ± 117	0.101
Troponin T, ng/L	5.0 [4.0–12.5]	5.0 [4.0–7.0]	0.363
NT-proB-type Natriuretic Peptide, pg/mL	119 [47–179]	58 [17–840]	0.325
Aspartate aminotransferase, U/L	42 [26–86]	53 [27–99]	0.795
Alanine transaminase, U/L	60 [37–122]	84 [41–206]	**0.035**
Gamma-glutamyl transferase, IU/L	162 [35–440]	245 [48–384]	0.945
Alkaline phosphatase, IU/L	85 [63–124]	94 [70–113]	0.698
Total bilirubin, mg/dL	0.5 [0.2–0.7]	0.4 [0.3–0.5]	0.655
Direct bilirubin, mg/dL	0.3 ± 0.2	0.3 ± 0.2	0.520
C peptide, ng/mL	7.8 ± 5.4	8.7 ± 4.8	0.572
Glycated hemoglobin, %	6.5 ± 1.0	6.6 ± 1.0	0.172
Glucagon, ng/L	223 ± 89	195 ± 48	0.224
**Echocardiography**			
Left ventricular ejection fraction, %	68 ± 3	69 ± 4	0.619
S wave, cm/s	16.6 ± 3.7	16.7 ± 6.5	0.946
Right ventricular fractional area change, %	38 [33–39]	40 [38–43]	0.936
Tricuspid annular plane systolic excursion, cm	21 ± 3	21 ± 3	0.311
Detectable tricuspid regurgitation velocity, *n* (%)	2 (15)	2 (15)	-

Data are presented as *n*, mean ± SD, or median [interquartile range].

**Table 3:** Safety endpoints at baseline and after five days of administration of Liraglutide in hospitalized COVID-19 patients presented by requirement for critical care admission (*n* = 12).

Laboratory Parameter	Non-Critical Care Cohort (*n* = 5)			Critical Care Cohort (*n* = 7)		
	Day 0	Day 5	*p*-Value	Day 0	Day 5	*p*-Value
Hemoglobin	13.92 ± 1.71	14.18 ± 1.52	0.452	14.00 ± 1.69	13.44 ± 1.31	0.460
Leukocytes	9461.67 ± 4073.95	9413.33 ± 2625.27	0.954	8782.86 ± 3330.04	9968.57 ± 2738.72	0.145
Neutrophils, %	83.67 ± 2.07	77.17 ± 16.67	0.341	87.57 ± 3.10	83.71 ± 6.97	0.240
Lymphocytes, %	10.00 ± 2.83	16.50 ± 14.73	0.261	8.71 ± 2.81	9.43 ± 4.08	0.733
Eosinophils, %	0.00 ± 0.00	0.62 ± 0.80	0.118	0.00 ± 0.00	0.41 ± 0.75	0.196
Platelets	321,166.67 ± 83,501.90	441,166.67 ± 153,231.09	0.014	253,857.14 ± 113,411.39	379,857.14 ± 176,538.41	0.029
Urea	51.33 ± 17.74	38.83 ± 7.52	0.062	50.00 ± 23.70	53.29 ± 19.53	0.622
Creatinine	0.89 ± 0.26	0.88 ± 0.09	0.921	0.95 ± 0.41	1.22 ± 1.14	0.397
Lactate dehydrogenase (LDH)	402.20 ± 128.87	321.80 ± 100.68	0.056	435.43 ± 120.24	470.71 ± 131.57	0.462
D-Dimer	0.98 ± 0.26	1.26 ± 0.44	0.346	1.08 ± 0.39	1.82 ± 1.29	0.205
Troponin (TNI), median|IQR	6.60 ± 3.13	4.60 ± 1.52	0.061	9.14 ± 6.52	57.86 ± 136.36	0.365
NTproBNP, median|IQR	159.02 ± 194.28	213.70 ± 397.90	0.622	147.90 ± 121.01	5221.90 ± 12,115.86	0.352
TGO-AST	84.80 ± 59.39	75.40 ± 58.52	0.733	45.14 ± 23.36	57.57 ± 35.33	0.340
TGP-ALT	124.00 ± 81.87	198.40 ± 152.63	0.138	57.00 ± 29.26	94.29 ± 74.63	0.194
Alkaline Phosphatase	125.60 ± 63.08	119.80 ± 61.46	0.465	78.57 ± 34.39	87.00 ± 22.06	0.397
Total Bilirubin	0.60 ± 0.27	0.35 ± 0.07	0.098	0.45 ± 0.26	0.54 ± 0.28	0.615
Fibrinogen	629.60 ± 76.76	546.40 ± 76.85	0.012	645.29 ± 87.01	608.43 ± 139.43	0.510
C peptide	9.97 ± 4.78	10.92 ± 2.54	0.752	6.30 ± 5.72	7.10 ± 5.51	0.668
Glycated hemoglobin HAB1C	6.80 ± 1.20	6.84 ± 1.04	0.717	6.26 ± 0.93	6.40 ± 0.93	0.182
Glucagon	200.00 ± 61.77	210.80 ± 47.54	0.634	238.86 ± 106.11	184.14 ± 49.25	0.183
**Echocardiography**						
Left ventricular ejection fraction (%)	67.60 ± 1.95	69.80 ± 2.28	0.004	69.20 ± 4.55	68.20 ± 5.81	0.669
S wave (cm/s)	14.68 ± 3.42	13.90 ± 3.85	0.763	19.67 ± 1.53	21.33 ± 8.08	0.707
RV FAC, %	35.75 ± 3.20	40.50 ± 3.11	0.064	44.00 ± 12.70	38.50 ± 5.20	0.572
TAPSE	21.40 ± 3.51	21.20 ± 3.49	0.704	20.50 ± 1.52	21.67 ± 2.50	0.201

**Table 4:** CD147 expression on lymphocytes and soluble plasma levels at baseline and after five days of administration of Liraglutide in hospitalized patients with COVID-19 pneumonia (*n* = 12).

	Day 0			Day 5		
Cytometry	Total (*n* = 12)	Non-Critical (*n* = 5)	Critical (*n* = 7)	Total (*n* = 12)	Non-Critical (*n* = 5)	Critical (*n* = 7)
B Lymphocyte, cells/mcL	7968.58 ± 2437.52	6981.40 ± 1951.64	8673.71 ± 2638.42	9252.83 ± 3900.71	8926.60 ± 2699.81	9485.86 ± 4783.70
T Lymphocyte, cells/mcL	4642.50 ± 986.24	4231.80 ± 689.21	4935.86 ± 1107.07	5262.00 ± 897.40 *	5450.80 ± 315.08 ^#^	5127.14 ± 1165.91
T Lymphocyte CD4, cells/mcL	4966.00 ± 1120.70	4423.40 ± 732.49	5353.57 ± 1234.64	5615.58 ± 991.30 *	5786.00 ± 423.50 ^#^	5493.86 ± 1280.83
T Lymphocyte CD8, cells/mcL	4070.58 ± 799.08	3831.00 ± 608.71	4241.71 ± 917.41	4731.00 ± 830.17 *	5049.00 ± 455.09 ^#^	4503.86 ± 990.44
NK Lymphocyte, cells/mcL	5353.92 ± 1451.53	4976.20 ± 959.51	5623.71 ± 1745.03	5744.67 ± 1155.53	5595.80 ± 1132.75	5851.00 ± 1249.36
**ELISA**						
CD147, pg/mL	5626.07 ± 1481.51	6139.30 ± 1408.60	5259.47 ± 1524.76	6090.68 ± 2854.90	4753.08 ± 974.43 ^#^	7046.10 ± 3428.34
MMP-2, ng/mL	144.97 ± 24.83	153.66 ± 22.75	138.76 ± 26.03	142.92 ± 15.08	146.96 ± 7.91	140.04 ± 18.76
MMP-9, ng/mL	219.71 ± 80.21	219.52 ± 70.27	219.85 ± 92.20	274.09 ± 138.30	354.10 ± 70.74 ^&^	216.94 ± 150.29
CD62E, ng/mL	32.94 ± 14.13	29.65 ± 9.26	35.29 ± 17.14	31.71 ± 12.20	30.06 ± 11.15	32.89 ± 13.64
**Interleukins**						
IL-6, pg/mL	46.04 [8.45–120.6]	22.27 [6.68–59.52]	84.94 [8.87–192.80]	6.77 [2.34–29.07] *	2.89 [2.13–3.79]	28.69 [9.48–55.60]
IL-8, pg/mL	25.44 ± 10.85	20.83 ± 7.56	28.74 ± 12.14	19.98 ± 24.13	8.71 ± 3.17 ^#^	28.03 ± 29.65
IL-10, pg/mL	4.82 [2.62–7.10]	3.15 [1.89–6.13]	5.50 [3.68–7.28]	1.77 [1.09–2.10] *	1.49 [1.10–1.77]	1.99 [1.00–3.48]

Data are presented as mean ± SD or median [interquartile range]. MMP: plasma matrix metallopeptidases. IL: interleukin. * *p* < 0.05 when comparing the total study sample between day 0 and day 5. ^#^ *p* < 0.05 when comparing non-critical patients between day 0 and day 5. ^&^ *p* = 0.05 when comparing non-critical patients between day 0 and day 5.

## Data Availability

Ethical restrictions limit accessibility of patient specific data related to this manuscript. Requests for patient specific data will be considered and provided at the discretion of the Authorship team.

## Supplementary Materials

The following supporting information can be downloaded at: https://www.mdpi.com/article/10.3390/idr17010005/s1, Table S1: online supplement): CD147 expression on lymphocytes and soluble plasma levels at baseline and after five days of administration of Liraglutide in hospitalized patients with COVID-19 pneumonia according to critical care requirement, Table S2: (Online supplement): Safety endpoints at baseline and after five days of administration of Liraglutide in hospitalized COVID-19 patients presented by requirement for critical care admission (*n* = 12). Table S3: (Online supplement): Raw patient data for patients receiving Liraglutide for acute COVID-19 pneumonia.
