# Supplementary material for: Early Use of Liraglutide for the Treatment of Acute COVID-19 Infection: An Open-Label Single-Center Phase II Safety Study with Biomarker Profiling
Source: Infect Dis Rep. 2025 Jan 10;17(1):5. doi: 10.3390/idr17010005 (PMC11755651; doi:10.3390/idr17010005)
Supplement: Supplementary file 1 [file idr-17-00005-s001.zip › idr-3303002-supplementary.pdf]

Table S1 (online supplement): CD147 expression on lymphocytes and soluble plasma levels at baseline and after five days of administration of Liraglutide in hospitalized patients with Covid-19 pneumonia according to critical care requirement.

	**Non-critical care (n=6)**	**Critical care (n=7)**	**p-value**	**Non-critical care (n=6)**	**Critical care (n=7)**	**p-value**
	**Day 0**			**Day 5**		
**Citometry**						
CD19+CD147+	7,682.17 ± 2,448.18	8,673.71 ± 2,638.42	0.500	8,926.60 ± 2,699.81	9,485.86 ± 4,783.70	0.820
CD3+CD147+	4,435.67 ± 793.34	4,935.86 ± 1,107.07	0.377	5,450.80 ± 315.08	5,127.14 ± 1,165.91	0.506
CD3+CD4+CD8-CD147+	4,551.83 ± 726.77	5,353.57 ± 1,234.64	0.191	5,786.00 ± 423.50	5,493.86 ± 1,280.83	0.590
CD3+CD4-CD8+CD147+	4,181.50 ± 1,016.62	4,241.71 ± 917.41	0.913	5,049.00 ± 455.09	4,503.86 ± 990.44	0.282
CD3-CD19-CD16/56+CD147+	5,110.83 ± 919.39	5,623.71 ± 1,745.03	0.532	5,595.80 ± 1,132.75	5,851.00 ± 1,249.36	0.725
**ELISA**						
CD147 pg/mL	5,843.37 ± 1,453.53	5,259.47 ± 1,524.76	0.497	4,753.08 ± 974.43	7,046.10 ± 3,428.34	0.181
MMP-2 ng/mL	153.28 ± 20.37	138.76 ± 26.03	0.293	146.96 ± 7.91	140.04 ± 18.76	0.460
MMP-9 ng/mL	227.62 ± 65.91	219.85 ± 92.20	0.867	354.10 ± 70.74	216.94 ± 150.29	0.090

Data is presented as mean ± SD or median [interquartile range]. MMP: plasma matrix metallopeptidases. IL: interleukin. * $p < 0.05$ comparing the total study sample between day 0 and day 5. # $p < 0.05$ comparing non-critical patients between day 0 and day 5. & $p = 0.05$ comparing non-critical patients between day 0 and day 5.

Table S2 (Online supplement): Safety endpoints at baseline and after five days of administration of Liraglutide in hospitalized Covid-19 patients presented by requirement for critical care admission (n=12).

	Non-critical care (n=6) Day 0	Critical care (n=7) Day 0	p-value Day 0	Non-critical care (n=6) Day 5	Critical care (n=7) Day 5	p-value Day 5
Age, years	58.50 ± 5.21	52.57 ± 10.50	0.24			
**Clinical status at admission**						
SpO2, %	93.67 ± 2.58	93.36 ± 2.56	0.83	93.00 ± 1.26	93.71 ± 1.80	0.43
Systolic blood pressure, mmHg	129.00 ± 16.79	119.29 ± 14.96	0.29	129.50 ± 20.13	130.71 ± 16.57	0.91
Diastolic blood pressure, mmHg	85.17 ± 18.30	77.00 ± 13.82	0.38	85.17 ± 8.61	74.43 ± 16.59	0.18
Heart rate, bpm	85.67 ± 25.57	81.86 ± 20.59	0.77	86.33 ± 9.11	94.14 ± 18.43	0.37
Respiratory rate, ipm	25.50 ± 6.57	27.29 ± 4.39	0.57	20.17 ± 1.60	23.00 ± 8.91	0.46
**Lab results**						
Haemoglobin, g/dL	13.92 ± 1.71	14.00 ± 1.69	0.93	14.18 ± 1.52	13.44 ± 1.31	0.37
Haematocrit, %	41.40 ± 3.78	41.73 ± 4.64	0.89	42.33 ± 3.21	39.89 ± 2.83	0.17
Leukocytes, count/mm3	9,461.67 ± 4,073.95	8,782.86 ± 3,330.04	0.75	9,413.33 ± 2,625.27	9,968.57 ± 2,738.72	0.72
Neutrophils, %	83.67 ± 2.07	87.57 ± 3.10	0.02	77.17 ± 16.67	83.71 ± 6.97	0.36
Lymphocytes, count/mm3	884.67 ± 267.79	714.14 ± 206.69	0.22	1,465.33 ± 1,148.80	989.71 ± 559.69	0.35
Lymphocytes, %	10.00 ± 2.83	8.71 ± 2.81	0.43	16.50 ± 14.73	9.43 ± 4.08	0.25
Eosinophils, %	0.00 ± 0.00	0.00 ± 0.00	-	0.62 ± 0.80	0.41 ± 0.75	0.65
Platelets, count/mcL	321,166.67 ± 83,501.90	253,857.14 ± 113,411.39	0.26	441,166.67 ± 153,231.09	379,857.14 ± 176,538.41	0.52
Urea, mg/dL	51.33 ± 17.74	50.00 ± 23.70	0.91	38.83 ± 7.52	53.29 ± 19.53	0.12
Creatinine, mg/dL	0.89 ± 0.26	0.95 ± 0.41	0.75	0.88 ± 0.09	1.22 ± 1.14	0.48
Sodium, mEq/L	134.33 ± 2.73	138.71 ± 2.21	0.01	133.67 ± 4.18	137.43 ± 4.93	0.17
Potassium, mEq/L	4.37 ± 0.47	4.19 ± 0.58	0.56	4.80 ± 0.64	4.67 ± 0.49	0.69
C-reactive protein, mg/L	68.85 ± 40.36	103.81 ± 34.54	0.12	19.22 ± 10.47	57.67 ± 42.72	0.06
Lactic dehydrogenase, U/L	382.00 ± 125.44	435.43 ± 120.24	0.45	321.80 ± 100.68	470.71 ± 131.57	**0.06**
D-Dimer, mg/L	0.97 ± 0.23	1.45 ± 1.04	0.30	1.26 ± 0.44	1.82 ± 1.29	0.39
Troponin T, ng/L	6.17 ± 2.99	9.14 ± 6.52	0.33	4.60 ± 1.52	57.86 ± 136.36	0.41
NT-proB-type Natriuretic Peptide, pg/mL	148.35 ± 175.73	143.77 ± 111.01	0.96	213.70 ± 397.90	4,478.63 ± 11,233.68	0.42
Aspartate aminotransferase, U/L	73.83 ± 59.53	45.14 ± 23.36	0.26	75.40 ± 58.52	57.57 ± 35.33	0.52
Alanine transaminase, U/L	113.33 ± 77.75	57.00 ± 29.26	0.10	198.40 ± 152.63	94.29 ± 74.63	0.15
Gamma-glutamyl transferase, IU/L	318.83 ± 291.48	185.43 ± 212.35	0.36	290.60 ± 267.02	224.00 ± 185.01	0.62
Alkaline phosphatase, IU/L	117.83 ± 59.54	78.57 ± 34.39	0.17	119.80 ± 61.46	87.00 ± 22.06	0.22
Total bilirubin, mg/dL	0.54 ± 0.28	0.45 ± 0.26	0.58	0.35 ± 0.07	0.54 ± 0.28	0.19
Direct bilirubin, mg/dL	0.34 ± 0.24	0.29 ± 0.17	0.68	0.20 ± 0.04	0.32 ± 0.23	0.27
C peptide, ng/mL	0.20 ± 0.12	0.16 ± 0.11	0.58	0.17 ± 0.05	0.21 ± 0.07	0.30
Fibrinogen, mg/dL	616.67 ± 75.62	645.29 ± 87.01	0.54	546.40 ± 76.85	608.43 ± 139.43	0.39
C peptide, ng/mL	10.06 ± 4.28	6.30 ± 5.72	0.21	10.92 ± 2.54	7.10 ± 5.51	0.18
Glycated haemoglobin, %	6.73 ± 1.08	6.26 ± 0.93	0.41	6.84 ± 1.04	6.40 ± 0.93	0.46
Glucagon, ng/L	198.67 ± 55.34	238.86 ± 106.11	0.42	210.80 ± 47.54	184.14 ± 49.25	0.37
**Echocardiography**						
Left ventricular ejection fraction, %	67.60 ± 1.95	70.57 ± 4.54	0.20	69.80 ± 2.28	68.20 ± 5.81	0.58
Tricuspid annular plane systolic excursion, cm	21.40 ± 3.51	21.00 ± 1.91	0.80	21.20 ± 3.49	21.67 ± 2.50	0.80
Right ventricular fractional area change, %	35.75 ± 3.20	40.33 ± 11.79	0.48	40.60 ± 2.70	38.50 ± 5.20	0.46
S wave, cm/s	14.68 ± 3.42	18.14 ± 3.19	0.14	13.90 ± 3.85	21.33 ± 8.08	0.12

Data is presented as n, mean ± SD or median [interquartile range].

Table S3 (Online supplement) : Raw patient data for patients receiving Liraglutide for acute Covid-19 pneumonia.

ID	Gender	Age	ICUadmission		SpO2roomair	SystolicarterialpressuremmHg	DyastolicarterialpressuremmHg	Heartratebpm	Respiratoryrateirpm
1.00	M	60.00	0.00	D0	94.00	144.00	91.00	69.00	20.00
				D5	91.00	116.00	85.00	99.00	20.00
2.00	M	58.00	0.00	D0	91.00	153.00	96.00	98.00	23.00
				D5	92.00	156.00	98.00	89.00	19.00
3.00	F	56.00	0.00	D0	96.00	119.00	77.00	68.00	18.00
				D5	94.00	111.00	80.00	86.00	20.00
4.00	F	53.00	0.00	D0	96.00	110.00	60.00	130.00	28.00
				D5	94.00	123.00	77.00	77.00	22.00
5.00	M	56.00	0.00	D0	95.00	131.00	112.00	87.00	36.00
				D5	94.00	117.00	78.00	92.00	22.00
6.00	M	68.00	0.00	D0	90.00	117.00	75.00	62.00	28.00
				D5	93.00	154.00	93.00	75.00	18.00
7.00	M	46.00	1.00	D0	88.00	128.00	86.00	79.00	29.00
				D5	92.00	152.00	97.00	93.00	21.00
8.00	F	66.00	1.00	D0	93.00	103.00	56.00	83.00	20.00
				D5	97.00	140.00	47.00	116.00	40.00
9.00	M	68.00	1.00	D0	93.50	128.00	72.00	50.00	29.00
				D5	94.00	147.00	83.00	64.00	27.00
10.00	M	46.00	1.00	D0	95.00	124.00	92.00	91.00	24.00
				D5	93.00	110.00	59.00	101.00	20.00
11.00	M	42.00	1.00	D0	96.00	120.00	84.00	63.00	28.00
				D5	95.00	115.00	76.00	76.00	17.00
12.00	M	46.00	1.00	D0	94.00	137.00	87.00	112.00	34.00
				D5	93.00	133.00	83.00	99.00	12.00
13.00	M	54.00	1.00	D0	94.00	95.00	62.00	95.00	27.00
				D5	92.00	118.00	76.00	110.00	24.00

ID	Gender	Age	ICUadmission		Hemoglobin	Hematocrit	leukocytes	Neutrophils	lymphocytesabsolut	lymphocytes	eosinophils	Platelets
1.00	M	60.00	0.00	D0	13.80	39.90	9,100.00	83.00	1,083.00	12.00	0.00	361,000.00
				D5	14.30	43.60	9,410.00	81.00	1,223.00	13.00	0.00	572,000.00
2.00	M	58.00	0.00	D0	13.80	41.90	7,220.00	85.00	729.00	10.00	0.00	442,000.00
				D5	15.30	45.10	9,410.00	83.00	1,167.00	12.00	0.70	658,000.00
3.00	F	56.00	0.00	D0	11.30	35.50	6,520.00	81.00	926.00	14.00	0.00	375,000.00
				D5	11.50	36.50	8,080.00	44.00	3,684.00	46.00	1.00	461,000.00
4.00	F	53.00	0.00	D0	13.10	40.40	15,970.00	83.00	1,278.00	8.00	0.00	266,000.00
				D5	13.40	40.80	13,840.00	81.00	1,522.00	11.00	2.00	391,000.00
5.00	M	56.00	0.00	D0	15.30	44.40	5,350.00	83.00	535.00	10.00	0.00	237,000.00
				D5	15.40	43.60	5,830.00	83.00	700.00	12.00	0.00	281,000.00
6.00	M	68.00	0.00	D0	16.20	46.30	12,610.00	87.00	757.00	6.00	0.00	246,000.00
				D5	15.20	44.40	9,910.00	91.00	496.00	5.00	0.00	284,000.00
7.00	M	46.00	1.00	D0	13.30	39.20	9,230.00	91.00	554.00	6.00	0.00	301,000.00
				D5	12.00	35.30	7,420.00	89.00	594.00	8.00	1.90	451,000.00
8.00	F	66.00	1.00	D0	14.40	43.10	7,100.00	88.00	788.00	11.00	0.00	138,000.00
				D5	12.40	38.20	8,040.00	93.00	249.00	3.00	0.00	285,000.00
9.00	M	68.00	1.00	D0	12.70	38.90	5,160.00	87.00	480.00	9.00	0.00	165,000.00
				D5	12.50	37.90	9,240.00	89.00	776.00	8.00	0.00	232,000.00
10.00	M	46.00	1.00	D0	17.30	51.40	5,490.00	83.00	675.00	12.00	0.00	138,000.00
				D5	13.80	42.70	8,380.00	84.00	670.00	8.00	0.00	158,000.00
11.00	M	42.00	1.00	D0	12.30	39.10	11,880.00	86.00	1,069.00	9.00	0.00	427,000.00
				D5	13.10	40.40	12,810.00	78.00	1,793.00	14.00	0.00	416,000.00
12.00	M	46.00	1.00	D0	14.70	42.20	14,240.00	92.00	570.00	4.00	0.00	253,000.00
				D5	14.90	42.40	14,760.00	74.00	1,476.00	10.00	0.00	423,000.00
13.00	M	54.00	1.00	D0	13.30	38.20	8,380.00	86.00	863.00	10.00	0.00	355,000.00
				D5	15.40	42.30	9,130.00	79.00	1,370.00	15.00	1.00	694,000.00

ID	Gender	Age	ICUadmission		Urea	Creatinine	SodiumNa	PotassiumK	PCRmgdL	laticdehydrogenaseDHL	DDimer	TroponinTNI
1.00	M	60.00	0.00	D0	29.00	0.74	135.00	4.10	61.30	525.00	0.99	6.00
				D5	31.00	0.88	136.00	4.90	20.80	330.00	0.62	4.00
2.00	M	58.00	0.00	D0	68.00	1.03	130.00	5.20	123.30	509.00	1.43	5.00
				D5	42.00	1.00	127.00	5.60	25.60	465.00	1.24	4.00
3.00	F	56.00	0.00	D0	33.00	0.53	136.00	4.30	30.70	281.00	0.92	4.00
				D5	34.00	0.78	137.00	4.00	10.00	-	-	-
4.00	F	53.00	0.00	D0	67.00	0.92	133.00	4.30	16.70	447.00	0.78	4.00
				D5	39.00	0.80	131.00	5.40	4.20	362.00	1.74	3.00
5.00	M	56.00	0.00	D0	46.00	0.81	138.00	4.50	92.10	253.00	0.84	6.00
				D5	35.00	0.83	138.00	4.70	32.90	226.00	1.58	5.00
6.00	M	68.00	0.00	D0	65.00	1.29	134.00	3.80	89.00	277.00	0.88	12.00
				D5	52.00	0.98	133.00	4.20	21.80	226.00	1.11	7.00
7.00	M	46.00	1.00	D0	30.00	0.84	137.00	4.40	120.60	663.00	1.55	5.00
				D5	45.00	0.74	141.00	4.10	92.00	586.00	3.58	6.00
8.00	F	66.00	1.00	D0	92.00	1.78	142.00	3.60	89.90	448.00	1.27	20.00
				D5	89.00	3.78	144.00	5.40	115.50	680.00	1.51	367.00
9.00	M	68.00	1.00	D0	59.00	1.15	140.00	4.50	171.30	389.00	1.13	13.00
				D5	66.00	0.90	140.00	4.10	100.10	540.00	1.80	13.00
10.00	M	46.00	1.00	D0	25.00	0.51	137.00	3.70	92.80	501.00	0.83	5.00
				D5	57.00	0.50	137.00	4.50	31.80	405.00	1.51	5.00
11.00	M	42.00	1.00	D0	58.00	0.86	137.00	5.00	63.80	287.00	3.71	14.00
				D5	47.00	0.97	129.00	5.10	18.60	327.00	1.51	7.00
12.00	M	46.00	1.00	D0	30.00	0.70	137.00	4.60	83.70	389.00	0.61	4.00
				D5	31.00	0.70	134.00	4.70	21.40	379.00	0.54	3.00
13.00	M	54.00	1.00	D0	56.00	0.82	141.00	3.50	104.60	371.00	1.02	3.00
				D5	38.00	0.94	137.00	4.80	24.30	378.00	1.34	4.00

ID	Gender	Age	ICUadmission		NTproBNP	TGOAST	TGPALT	GGT	AlkalinePhosphatase	TotalBilirrubin	directbilirrubin	indirectbilirrubin
1.00	M	60.00	0.00	D0	201.40	163.00	161.00	614.00	138.00	0.91	0.76	0.15
				D5	35.50	56.00	165.00	384.00	106.00	0.30	0.20	0.10
2.00	M	58.00	0.00	D0	64.70	124.00	229.00	742.00	227.00	0.57	0.38	0.19
				D5	58.00	167.00	379.00	702.00	225.00	0.41	0.25	0.16
3.00	F	56.00	0.00	D0	95.00	19.00	60.00	162.00	79.00	0.23	0.10	0.13
				D5	-	-	-	-	-	-	-	-
4.00	F	53.00	0.00	D0	15.00	77.00	140.00	95.00	65.00	0.22	0.14	0.08
				D5	17.00	96.00	333.00	91.00	75.00	0.26	0.14	0.22
5.00	M	56.00	0.00	D0	33.00	44.00	74.00	266.00	113.00	0.78	0.36	0.42
				D5	33.00	39.00	86.00	240.00	116.00	0.40	0.23	0.17
6.00	M	68.00	0.00	D0	481.00	16.00	16.00	34.00	85.00	0.50	0.27	0.23
				D5	925.00	19.00	29.00	36.00	77.00	0.39	0.19	0.20
7.00	M	46.00	1.00	D0	132.60	45.00	87.00	624.00	134.00	0.78	0.53	0.25
				D5	396.70	35.00	60.00	493.00	114.00	0.42	0.30	0.12
8.00	F	66.00	1.00	D0	156.60	24.00	25.00	23.00	40.00	0.18	0.11	0.07
				D5	29,945.00	68.00	61.00	49.00	68.00	1.09	0.77	0.32
9.00	M	68.00	1.00	D0	376.40	40.00	49.00	142.00	61.00	0.21	0.16	0.05
				D5	839.70	108.00	213.00	383.00	111.00	0.30	0.18	0.12
10.00	M	46.00	1.00	D0	41.80	95.00	103.00	192.00	90.00	0.54	0.38	0.16
				D5	17.00	99.00	186.00	315.00	87.00	0.70	0.42	0.28
11.00	M	42.00	1.00	D0	127.00	42.00	56.00	32.00	68.00	0.70	0.33	0.37
				D5	15.00	24.00	35.00	32.00	64.00	0.34	0.14	0.20
12.00	M	46.00	1.00	D0	119.00	42.00	54.00	249.00	110.00	0.57	0.42	0.15
				D5	19.00	49.00	81.00	249.00	101.00	0.57	0.35	0.22
13.00	M	54.00	1.00	D0	53.00	28.00	25.00	36.00	47.00	0.17	0.08	0.09
				D5	118.00	20.00	24.00	47.00	64.00	0.33	0.11	0.22

ID	Gender	Age	ICUadmission		Fibrinogen	Cpeptide	glycatedhemoglobinHAB1C	Glucagon
1.00	M	60.00	0.00	D0	577.00	2.76	6.10	129.00
				D5	552.00	11.45	6.30	222.00
2.00	M	58.00	0.00	D0	729.00	8.35	8.70	158.00
				D5	666.00	10.43	8.40	140.00
3.00	F	56.00	0.00	D0	552.00	10.50	6.40	192.00
				D5	-	-	-	-
4.00	F	53.00	0.00	D0	537.00	12.70	7.10	244.00
				D5	455.00	11.67	7.10	244.00
5.00	M	56.00	0.00	D0	678.00	15.32	5.60	189.00
				D5	543.00	7.02	5.60	189.00
6.00	M	68.00	0.00	D0	627.00	10.73	6.50	280.00
				D5	516.00	14.02	6.80	259.00
7.00	M	46.00	1.00	D0	737.00	2.88	6.50	245.00
				D5	799.00	3.26	6.50	152.00
8.00	F	66.00	1.00	D0	495.00	19.00	6.60	259.00
				D5	706.00	16.02	6.70	270.00
9.00	M	68.00	1.00	D0	757.00	5.42	5.90	171.00
				D5	727.00	3.02	6.20	148.00
10.00	M	46.00	1.00	D0	649.00	5.71	5.40	455.00
				D5	445.00	6.70	5.80	209.00
11.00	M	42.00	1.00	D0	604.00	2.69	5.50	235.00
				D5	445.00	13.45	5.20	162.00
12.00	M	46.00	1.00	D0	645.00	4.39	5.80	178.00
				D5	577.00	5.44	6.20	216.00
13.00	M	54.00	1.00	D0	630.00	4.00	8.10	129.00
				D5	560.00	1.81	8.20	132.00
